# Serotonin transporter gene polymorphisms and brain function during emotional distraction from cognitive processing in posttraumatic stress disorder

**DOI:** 10.1186/1471-244X-11-76

**Published:** 2011-05-05

**Authors:** Rajendra A Morey, Ahmad R Hariri, Andrea L Gold, Michael A Hauser, Heidi J Munger, Florin Dolcos, Gregory McCarthy

**Affiliations:** 1 Department of Psychiatry and Behavioral Sciences, Duke University, Durham, NC 27710 USA; 2 Duke-UNC Brain Imaging and Analysis Center, Duke University, Durham, NC 27705 USA; 3 Mid-Atlantic Mental Illness Research Education and Clinical Center, Durham VA Medical Center, Durham, NC 27705 USA; 4 Department of Psychology & Neuroscience, and Institute for Genome Sciences and Policy, Duke University, Durham, NC 27708 USA; 5 Department of Psychology, Yale University, New Haven, CT 06520 USA; 6 Center for Human Genetics, Duke University, Durham, NC 27710 USA; 7 Department of Psychology, Neuroscience Program, and Beckman Institute for Advanced Science & Technology, University of Illinois, Urbana-Champaign, IL, USA

**Keywords:** PTSD, imaging genetics, ventrolateral PFC, amygdala, *SLC6A4*, rs16965628, working memory, emotion processing, cognitive control

## Abstract

**Background:**

Serotonergic system dysfunction has been implicated in posttraumatic stress disorder (PTSD). Genetic polymorphisms associated with serotonin signaling may predict differences in brain circuitry involved in emotion processing and deficits associated with PTSD. In healthy individuals, common functional polymorphisms in the serotonin transporter gene (*SLC6A4*) have been shown to modulate amygdala and prefrontal cortex (PFC) activity in response to salient emotional stimuli. Similar patterns of differential neural responses to emotional stimuli have been demonstrated in PTSD but genetic factors influencing these activations have yet to be examined.

**Methods:**

We investigated whether *SLC6A4 *promoter polymorphisms (5-HTTLPR, rs25531) and several downstream single nucleotide polymorphisms (SNPs) modulated activity of brain regions involved in the cognitive control of emotion in post-9/11 veterans with PTSD. We used functional MRI to examine neural activity in a PTSD group (n = 22) and a trauma-exposed control group (n = 20) in response to trauma-related images presented as task-irrelevant distractors during the active maintenance period of a delayed-response working memory task. Regions of interest were derived by contrasting activation for the most distracting and least distracting conditions across participants.

**Results:**

In patients with PTSD, when compared to trauma-exposed controls, rs16965628 (associated with serotonin transporter gene expression) modulated task-related ventrolateral PFC activation and 5-HTTLPR tended to modulate left amygdala activation. Subsequent to combat-related trauma, these *SLC6A4 *polymorphisms may bias serotonin signaling and the neural circuitry mediating cognitive control of emotion in patients with PTSD.

**Conclusions:**

The *SLC6A4 *SNP rs16965628 and 5-HTTLPR are associated with a bias in neural responses to traumatic reminders and cognitive control of emotions in patients with PTSD. Functional MRI may help identify intermediate phenotypes and dimensions of PTSD that clarify the functional link between genes and disease phenotype, and also highlight features of PTSD that show more proximal influence of susceptibility genes compared to current clinical categorizations.

## Background

Imaging genetics has been used to identify the role of genes in modulating brain differences associated with behavioral and cognitive symptom features in a number of psychiatric disorders [1,2], including mood disorders [3], anxiety disorders [4-6], and schizophrenia [7]. Whereas imaging genetics has generally relied on exploration of candidate gene effects, gene discovery has generally been accomplished through genome wide association studies (GWAS). Recently, however, imaging genetics has become a fruitful avenue for gene discovery or identifying allelic variants of known candidate genes that are associated with brain disorders such as schizophrenia and Alzheimer's disease [8,9]. Neuroimaging studies have revealed important structural and functional brain abnormalities in neuropsychiatric disorders, and mounting evidence suggests that genetic variability is reflected in brain activity as observed with neuroimaging methods [10].

Although imaging genetics studies in PTSD are lacking, a few studies have examined candidate gene associations with the behavioral phenotype of PTSD [11-17]. These studies are consistent with evidence of a genetically mediated vulnerability to PTSD in the context of traumatic stress exposure. Individuals with a family history of PTSD have a 3 to 5 fold relative risk of developing PTSD [18-20], and twin studies suggest the heritability for PTSD is over 30% [21,22]. Growing evidence suggests that the serotonin transporter gene linked polymorphic region (5-HTTLPR) is associated with risk for PTSD. The frequency of 5-HTTLPR short homozygotes was greater in PTSD patients relative to healthy control subjects who were not recruited for the presence of trauma exposure [11]. Recent studies have begun to examine the role of gene-environment interplay in PTSD risk mechanisms [23]. In a study of highly traumatized Rwandan refugees, 5-HTTLPR genotype predicted PTSD risk; individuals homozygous for the short allele were at high risk for developing PTSD regardless of levels of trauma exposure, whereas the other genotypes exhibited a dose-response relationship of the number of lifetime trauma events with risk for PTSD [14]. In hurricane survivors, the expression levels of the serotonin transporter gene were associated with PTSD, but only in the setting of high exposure to stress and low social support [12]. A recent study showed the 5-HTTLPR genotype alone did not predict PTSD, but rather interacted with childhood adversity and adult traumatic events to increase the risk of PTSD, particularly with high levels of exposure to both trauma types [15].

However, the "common disease common variants" hypothesis suggests multiple genes and gene variants (common variants) are likely to influence risk for PTSD (common disease) [24], and therefore these initial intriguing associations are likely only a small part of the story. The effect of individual gene variants may be more precisely characterized by examining phenotypes closer to the biological activity of the gene in the context of PTSD [25,26]. Imaging genetics is one approach that is gaining interest in the assessment of the genetic modulation of neural activity associated with specific behavioral phenotypes [1,26,27]. For instance, variants of the 5-HTTLPR gene have been associated with differential activation in the amygdala [28-35], a region of the brain associated with fear learning [36] and shown to be hyperactive during emotion processing in PTSD [37,38]. For example, patients with PTSD exhibited greater activation for trauma-relevant pictures in the amygdala and ventrolateral prefrontal cortex (PFC), when compared to trauma-exposed controls [37,39-41]. Furthermore, *SLC6A4 *variants have been linked to alterations in prefrontal activation during cognitive processing [28]. The inferior PFC, particularly the ventrolateral PFC, plays an important role in the cognitive processing of emotionally salient information [42-47]. Genetic mechanisms appear to influence serotonergic pathways related to human fear conditioning [48]. Fear conditioning models have been applied to prominent PTSD symptoms (e.g., hypervigilance and exaggerated fear response to cues of the traumatic event) and proposed to inform neuroimaging and genetics investigations of PTSD (reviewed in [49,50]).

Our goal was to investigate the link between common variants of the serotonin transporter gene (including 5-HTTLPR) and known functional brain differences in PTSD. Our hypotheses followed from the previously demonstrated role of this candidate gene in modulating neural activity in emotion processing regions [51] coupled with the findings of genetic influences in PTSD [11-14] and other anxiety disorders [52,53].

Until recently, the 5-HTTLPR was analyzed as functionally biallelic with Long (L, 16 repeats) and Short (S, 14 repeats) alleles where the S allele leads to lower expression of mRNA and reduced serotonin transporter in membranes. More recently a functionally triallelic classification includes an A/G single nucleotide polymorphism (SNP), rs25531, that is observed predominantly within the L allele (L_A _and L_G _alleles)[54,55]. In light of reported effects of intragenic SNPs on transcriptional activity, it is important to evaluate not only these promoter polymorphisms, but common sequence variation across the entire gene for association with altered brain function in PTSD. We predicted that variation in the following functional polymorphisms would modulate neural activity in the amygdala and the ventrolateral PFC in patients with PTSD: 5-HTTLPR/rs25531 previously associated with severity of PTSD, rs140701 previously associated with panic disorder [52], and rs16965628 previously associated with obsessive compulsive disorder (OCD) in haplotype analysis [53] and recently reported to exert the greatest relative effect of common *SLC6A4 *variants on serotonin transporter gene expression in human cell lines [56]. Across all variants, we hypothesized that those previously associated with PTSD or other anxiety disorders and/or a relative decrease in 5-HTT expression would predict increased amygdala and ventrolateral PFC activation [37,40,41,57,58]. Moreover, consistent with GxE models we hypothesized that any genotype-related differences in brain function would be most pronounced in patients with PTSD.

In a previously reported fMRI study of PTSD [37], we assessed neural activation in a working memory task during the delay interval between encoding and retrieval when active maintenance of visual information was disrupted by the presentation of trauma-related distractors. The present study examined the role of serotonin transporter gene variants on PTSD patients and trauma-exposed controls whose regional brain activity we previously reported [37].

## Subjects and Methods

Here, we provide details of genotyping and statistical analyses of candidate gene effects on fMRI data. Detailed information about participants, cognitive challenge task, and fMRI analyses are previously published [37] and repeated here in summary form.

### Participants

Participants included a PTSD group (n = 22) and a trauma-exposed control group (n = 20) with comparable levels of combat exposure measured by the Combat Exposure Scale [t(40) = 1.2, p = 0.2]. Subjects provided written informed consent to participate in procedures approved by the Institutional Review Boards at Duke University and Durham VA Medical Center. The 42 subjects who underwent fMRI assessment were genotyped as part of a *parent sample *of 387 registry subjects. Subjects completed a screening battery to assess comorbid neuropsychiatric disorders (see Table 1). The Davidson Trauma Scale (DTS) was administered just prior to scanning to assess PTSD symptom severity (Davidson et al. 1997). Lacking a diagnostic interview in these subjects, a DTS cutoff score of 32, previously shown by us to have high diagnostic efficiency (0.94) in the post-9/11 military cohort [59], was used to divide the participants into a PTSD group with mean DTS (SD) = 74.4 (18.8) and Control group with mean DTS = 10.2 (8.8). The use of two diagnostic groups in favor of a correlational approach was further influenced by the presence of a strong bimodal distribution of DTS scores.

**Table 1:** Demographic and Clinical Characteristics of Subject Sample^1^

Characteristic	Control *n *= 20	PTSD *n *= 22	Group Comparison
Age (years) [SD]	37.6 [11.0]	30.8 [8.8]	t(40) = 2.2, *p *< 0.05

Gender, No.(%) of females	7 (35.0)	13 (59.1)	χ^**2**^(1) = .29, *p *> 0.5

Handedness, No.(%) right-handed	17 (85.0)	19 (86.4)	χ^**2**^(2) = 0.68, *p *> 0.7

Race, No.(%) of Caucasian subjects	8 (40.0)	12 (54.5)	χ^**2**^(2) = 2.1, *p *> 0.3

Education (years) [SD]	13.9 [2.8]	13.3 [1.8]	t(40) = 0.8, *p *> 0.4

Davidson Trauma Scale [SD]	10.2 [8.8]	74.4 [18.8]	t(40) = 13.9, *p *< 0.001

Combat Exposure Scale [SD]	8.6 [11.0]	12.6 [10.3]	t(40) = 1.2, *p *> 0.2

Beck Depression Inventory [SD]	7.1 [6.1]	20.8 [9.0]	t(40) = 5.7, *p *< 0.001

Alcohol Use Disorders Identification Test [SD]	2.6 [3.2]	6.1 [6.3]	t(40) = 2.6, *p *< 0.05

Drug Abuse Screening Test, [SD]	0.4 [0.8]	2.1 [2.5]	t(40) = 2.9, *p *< 0.01

Antidepressant Medication, No. (%) prescribed ^**2**^	1 (5.0)	8 (36.4)	χ^**2**^(1) = 6.1, *p *< 0.01

Antidepressant Dosage equivalents [SD] ^**2**^	0.9 [4.3]	14.5 [19.9]	t(40) = 3.0, *p *< .005

^**1 **^Data values represent means except where indicated otherwise.

^**2 **^Antidepressant medications taken were either selective serotonin reuptake inhibitors (SSRIs) or mirtazipine. Antidepressant dosage equivalents are listed in Table 3.

### Genotyping Methods

**Figure 1:**
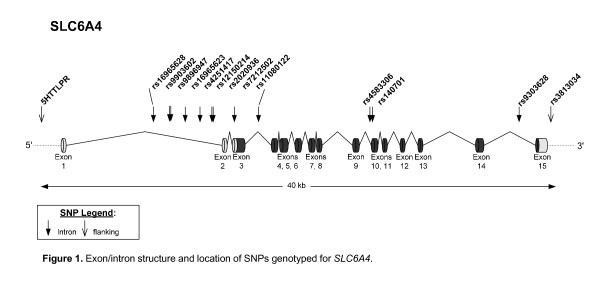
**Exon/intron structure and location of SNPs genotyped for *SLC6A4***. The SNP rs16965628 previously associated with obsessive compulsive disorder (OCD) exerts the greatest relative effect of common SLC6A4 variants on serotonin transporter gene expression in human cell lines.

SNPs for *SLC6A4 *(see Figure 1) were chosen using phase II Caucasian (CEU) and Yoruban (YRI) genotype data of the International HapMap Project [60]. A combined list of tagging SNPs was selected with LD-Select Version 1.0 [61] and MultiPop-TagSelect Version 1.1 software [62], with r^2 ^= 0.3 and minor allele frequency (MAF) > 0.1. Coding SNPs with MAF > 0.1 were forced into the list. SNPs were genotyped using TaqMan^® ^SNP Genotyping Assays (Applied Biosystems Inc.). The 5-HTTLPR/rs25531 polymorphism was genotyped in two parts. After PCR amplification, 1 μl of product was used for fragment analysis of the short (S), long (L), and extra long (XL) alleles of the insertion/deletion polymorphism (484, 528, and 594 bp, respectively; ABI 3730 DNA Analyzer Capillary Array; GeneMapper^® ^Software, version 4.0, Applied Biosystems Inc.). The remaining product was digested by restriction enzyme HpaII (New England BioLabs Inc) to determine the L_G_, S (S_A_) and L_A _alleles (174, 297 and 340 bp, respectively). Call rates for all polymorphisms analyzed in this study were ≥95%.

### Stimuli and Working Memory Task Design

During the fMRI scan, subjects performed a working memory task with combat-related and control distractors. Each trial consisted of an encoding phase, a delay period with trauma-related and non-trauma-unrelated visual distractor scenes and a retrieval phase for an epoch duration of 29 s with 12.5 s between epochs. The encoding phase consisted of three similar faces presented for 3.5 s, which subjects encoded into working memory and maintained for 11.5 s. The visual distractors consisted of two consecutively displayed (i) combat scenes, (ii) non-combat scenes, or (iii) digitally scrambled pictures (control condition) presented for 3 s each. Combat and non-combat scenes were adapted from a superset of images [58] for which combat scenes had more negative emotional valence than non-combat scenes. During the retrieval phase, a single-face was presented requiring a button response to indicate its presence (old) or absence (new) during encoding. Subjects viewed 40 trials per stimulus type.

### Imaging Protocol

Images were acquired on a 4T General Electric SIGNA MRI scanner. Full-brain coverage was obtained with 34 interleaved axial functional slices (TR/TE/flip = 2000 ms/31 ms/60°; FOV = 240 mm; 3.75 × 3.75 × 3.8 mm voxels; interslice skip = 0) using an inverse-spiral pulse sequence. High-resolution 3D spin-echo co-planar structural images were acquired in 68 axial slices (TR/TE/flip = 12.2 ms/5.3 ms/20°, voxel size = 1 × 1 × 1.9 mm, FOV = 240 mm, interslice skip = 0).

### Analysis of Functional MRI Data

Preprocessing of individual functional data sets was performed with FSL version 3.3.5 [Oxford Centre for Functional Magnetic Resonance Imaging of the Brain (FMRIB), Oxford University, U.K.][63]. All registrations were carried out using FMRIB Linear Image Registration Tool (FLIRT) for linear (affine with 12 degrees of freedom) registration [64]. Following preprocessing, subsequent data analyses used whole brain voxel-wise and region of interest (ROI) approaches to compare brain activity associated with the contrasts of interest (e.g., combat vs. non-combat conditions). For individual subject analyses, the fMRI signal was selectively averaged in each subject as a function of trial type (i.e., combat, non-combat, and scrambled) and image volume (TR) within the trial epoch (two image volumes preceding epoch onset and 14 image volumes following epoch onset), and compared for the contrasts of interest using pairwise t-statistics. Individual subject analyses produced whole-brain average and activation t-maps for each condition, contrast of interest, and sub-epochs (encoding, maintenance, and retrieval). Data for sub-epochal contrast maps was extracted from the overall time course by averaging image volumes representing maximal change relative to the pre-memorandum onset baseline.

### Determination of Functional Regions of Interest

**Figure 2:**
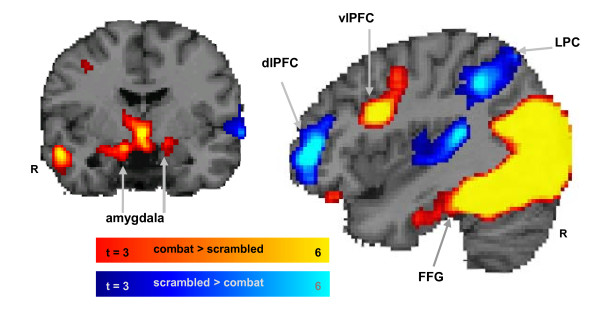
**Definition of functional regions of interest**. Five functional ROIs were defined from dissociable dorsal-ventral patterns of activity observed during the working memory delay period (in the presence of distractors in 42 subjects). The most disruptive effect on activity during the delay period in a set of dorsal brain regions associated with working memory (blue blobs) including the dorsolateral PFC (dlPFC) and the lateral parietal cortex (LPC). Combat distracters produced the most enhancing effect on activity on ventral brain regions associated with emotion processing (red blobs) including the amygdala, ventrolateral PFC, and fusiform gyrus. The activation maps show direct contrasts between the most versus least distracting conditions, combat > scrambled (red) and scrambled > combat (blue), with colored gradient bars indicating t values.

Functional regions of interest (ROIs) were defined by voxels showing the maximum effects during the active maintenance period for the contrasts of interest (see Figure 2). Specifically, contrast activation maps between the most vs. least distracting conditions (combat > scrambled distractors) showed strong activation in the amygdala, ventrolateral PFC, and fusiform gyrus, but the inverse contrast (scrambled > combat distractors) showed strong deactivations (signal activity below the inter-trial baseline) in the dorsolateral PFC (dlPFC) and lateral parietal cortex (LPC). Given our *a priori *hypotheses derived from non-clinical participants [45], we used an intensity threshold of *t *> 3.0 (*p *< 0.002) and an extent threshold of 10 contiguous voxels solely for the purpose of defining functional ROIs.

### Statistical Analyses

Analysis of working memory performance, as measured by detectability scores (d-prime = |Z(hit rate) - Z(false alarm rate)|) and defined by standard signal detection theory, used general linear modeling (GLM) to determine the influence of genotype. The dependent variable was repeated-measures d-prime scores for distractor-type (combat distractor, non-combat distractor scene). The factors and covariates were identical to fMRI data analysis described below.

The main hypothesis was tested by interrogating the genetic modulation of neural activity in PTSD relative to trauma-exposed control subjects. The dependent measure in the GLM was the difference in mean activation (percent signal change) between combat distractors and non-combat distractor scenes during the active maintenance phase of the working memory task in the functional ROIs. Factors were *diagnosis *(2 levels; PTSD, control) and *genotype *with the number of levels specific to the genetic polymorphism being tested. For example, 3 levels associated with transcriptional efficiency were used for triallelic 5-HTTLPR (low, reference, high) and 2 levels for rs16965628 (CG, GG). The number of levels per genotype factor are listed in Table 2 for each polymorphism. Higher depression scores and antidepressant medication usage in the PTSD group prompted the introduction of two covariates, score on the Beck Depression Inventory (BDI; [65]), and antidepressant medication dosage equivalents (see Table 3). There were no significant differences in trauma exposure as measured by the Combat Exposure Scale ([66]; (see Table 1). Despite no significant differences in the means and variances of ROI activation between African American and European American subjects (see Table 4, race was included as a covariate to account for the considerable differences in allele frequencies between individuals of African and European ancestry. Finally, to assess the possibility of stratification due to race we carried out a race based analysis for the significant results. Despite the low sample size in the resulting cells, main effect of genotype, then an effect of genotype within diagnostic groups, was assessed to confirm a pattern in the same direction as the overall finding.

**Table 2:** Allele and genotype frequencies for SLC6A4 polymorphisms

polymorphism	genotypes	genotype sample size	**group sizes [control,PTSD]**^**4**^	minor allele frequency	**HWE Χ**^**2**^	p-value	include genotypes
rs9903602	GG; GT; TT	6, 9, 27	2,4; 3,6; 12,15	.178	7.7	.005	exclude

rs9896947	CC, CT, TT	28, 14, 0	13,15; 7,7; 0,0	.833	1.68	.20	CC,CT

rs9303628	AA; AG;GG	6, 16, 20	3,3; 10,6; 7,13	.333	.86	.35	AG, GG

rs7212502	AA; AG; GG	38, 4, 0	20,18; 2,2; 0,0	.952	.105	.75	exclude

rs4583306	AA; AG; GG	17, 18, 7	10,7; 7,11; 3,4	.619	.350	.55	AA, AG

rs4251417	CC; CT; TT	36, 6, 0	18,18; 2,4; 0,0	.929	.25	.68	exclude

rs3813034	AA; AC; CC	14, 16, 12	9,5; 7,9; 4,8	.274	2.34	.13	exclude

rs2020936	AA, AG, GG	23, 17, 2	9,14; 10,7; 1,1	.750	.265	.61	AA, AG

rs16965628 ^**3**^	CC; CG; GG	0, 15, 27	0,0; 8,7; 13,14	.179	1.98	.18	CG, GG

rs16965623	AA; AG; GG	37, 5, 0	18,19; 2,3; 0,0	.940	.170	.68	exclude

rs140701 ^**3**^	CC; CT; TT	11, 21, 10	7,4; 8,13; 5,6	.512	.000	.99	CT, TT

rs12150214	CC; CG; GG	3, 17, 22	1,2; 11,16; 8,14	.274	.013	.91	CG, GG

rs11080122	CC; CT; TT	26, 16, 0	10,16; 10,6; 0,0	.810	2.33	.13	CC, CT

triallelic 5-LTTLPR ^**3**^	SS,SL_G_,L_G_L_G_; SL_A_,L_G_L_A_; L_A_L_A_	15, 18, 9	10,5; 6,12; 6;3	.571	.656	.42	SS,SL_G_,L_G_L_G_; SL_A_,L_G_L_A_;

biallelic 5-HTTLPR ^**3**^	SS; SL; LL	10, 20, 12	3,7; 12,8; 5,7	.476	.087	.77	S carriers, LL

^**3 **^polymorphisms with a priori hypothesis

^**4 **^group sizes are reported the number of control and PTSD subjects for each of three genotypes, listed in the order [homozygous, heterozygous, homozygous] and secondarily alphabetical order of coding bases (A, C, G, T).

Abbreviations: Hardy-Weinberg Equilibrium (HWE)

**Table 3:** Medication dose and dose equivalents

Subject	Group	Medication(s)	**Dose Equivalents **^**7**^
1	Control	mirtazipine 15 mg	20

2	PTSD	sertraline 50 mg, fluoxetine 10 mg	30

3	PTSD	paroxetine 40	40

4	PTSD	sertraline 100	40

5	PTSD	mirtazipine 15 mg, citalopram 10	30

6	PTSD	mirtazipine 15	20

7	PTSD	mirtazipine 15 mg, sertraline 100	60

8	PTSD	sertraline 50 mg	20

9	PTSD	mirtazipine 30	40

^**7 **^Antidepressant medication dosage equivalents based on the following dose equivalence formula: 20 mg citalopram = 50 mg sertraline = 5 mg escitalopram = 50 mg fluvoxamine = 20 mg paroxetine = 20 mg fluoxetine = 15 mg mirtazipine.

**Table 4:** Effect of race on mean ROI activation

	main effect race, race * genotype (p-value; uncorrected)				
	
Polymorphism	vlPFC	amygdala	fusiform gyrus	dlPFC	LPC
rs16965628	.13, .34	.26, .02	.39, .45	.55, .57	.53, .63

rs9896947	.25, .64	.32, .14	.07, .05	.33, .24	.30, .27

rs9303628	.44, .84	.91, .94	.35, .81	.55, .58	.69, .22

rs4583306	.01, .13	.12, .25	.45, .47	.42, .13	.15, .21

rs2020936	.30, .75	.91, .06	.16, .93	.69, .15	.52, .94

rs140701	.22, .98	.14, .25	.38, .87	.33, .03	.42, .32

rs12150214	.32, .80	.67, .17	.46, .88	.59, .30	.44, .99

rs11080122	.54, .81	.98, .32	.19, .68	.71, .19	.68, .77

triallelic 5HTTLPR	.13, .66	.67, .21	.34, .66	.54, .38	.96, .05

biallelic 5HTTLPR	.15, .46	.75, .84	.51, .81	.88, .26	.74, .04

Given the concern of statistical power with a small sample size, we considered only polymorphisms that had a minimum of six subjects per genotype, or five subjects for polymorphisms with *a priori *hypotheses. Accordingly for biallelic 5-HTTLPR, the genotypes were categorized as S allele carriers (SS, SL) or non-S allele carriers (LL) to enable analysis of 5 subjects per genotype category. For triallelic 5-HTTLPR, the low expressing group included genotypes L_G_L_G_, SL_G_, SS, the reference group included genotypes SL_A _and L_G_L_A _genotypes, and the high expressing genotype (L_A_L_A_) was not included due to inadequate sample (see Table 2). Sample size information on the remaining polymorphisms is provided in Table 2. Based on this restriction, only nine (8 SNPs + 1 promoter) of the 14 polymorphisms assayed on *SLC6A4 *were considered in the final analysis. All polymorphisms were tested for Hardy-Weinberg disequilibrium in the PTSD and control groups and by race in the parent sample and separately in the present sample. The SNPs that significantly modulated neural activity in hypothesized ROIs were assessed for LD with the 5-HTTLPR.

Adjustments for multiple comparisons were made with the Nyholt correction for testing multiple SNPs in linkage disequilibrium (LD) based on the spectral decomposition of matrices of pairwise LD between SNPs [67]. The Nyholt correction reduced the number of effective comparisons from eight to six. The 5-HTTLPR polymorphism was considered an independent test. Thus, to maintain the Type I error rate at .05 given a total of 7 effective comparisons (6+1), the p-values were multiplied by 7. The reported p-values are from the omnibus F-test for a GLM that includes genotype, PTSD status and genotype*diagnosis product (interaction) term. Only when the corrected p-value (p_corr_) for the omnibus F-test was significant (p_corr _< .05), did we report specific p-values for main effect of genotype and the interaction genotype*diagnosis. The main effects for diagnosis were reported in detail previously [37].

## Results

### Working memory performance

Working memory performance was measured using detectability scores (d-prime or D') and tested with a GLM. No significant effects were found for *SLC6A4 *polymorphisms rs16965628 [F(1,36) = .83, p = .37], triallelic 5-HTTLPR [F(1,34) = .61, p = .44], or rs140701 [F(1,34) = 2.2, p = .15]. Behavioral results for the remaining polymorphisms are summarized in Table 5.

**Table 5:** SLC6A4 and PTSD effects on mean ROI activation and working memory performance^5^

	p-value(corrected)					
	
Polymorphism	ventrolateral PFC	amygdala	fusiform gyrus	dlPFC	LPC	D'
rs16965628	.03*, .05*, .03*	.89	.57	.22	.45	.99

rs9896947	.19	.99	.44	.99	.24	.99

rs9303628	.60	.25	.10,	.18	.24	.99

rs4583306	.40	.64	.54	.78	.93	.99

rs2020936	.88	76	.77	.99	.67	.77

rs140701	.99	.99	.34	.99	.99	.99

rs12150214	.79	.87	.76	.99	.95	.69

rs11080122	.09	.98	.51	.99	.24	.99

triallelic 5HTTLPR	.18	.95	.16	.99	.99	.99

biallelic 5HTTLPR	.51	**.34 **^**6**^	.14	.99	.99	.99

^**5 **^The p-value(s) are from the omnibus F-test for a general linear model (GLM) that includes genotype, diagnosis (PTSD status) and genotype*diagnosis product (interaction) term. Factors include diagnosis (2 levels; PTSD, control) and genotype with 2 or 3 levels (see Table 2). Covariates are race (African American or European American), score on the Beck Depression Inventory (BDI; see Table 1) and antidepressant medication dose equivalents (see Table 3). Significance level was adjusted by multiplying by the number of effective multiple comparisons (seven) that was calculated with the Nyholt correction.

^**6 **^left amygdala (p_corr _= .07), right amygdala (p_corr _>.2).

Abbreviations: region of interest (ROI), dorsolateral prefrontal cortex (dlPFC), lateral parietal cortex (LPC).

### Genetic polymorphisms in SLC6A4 and neural activation

**Figure 3:**
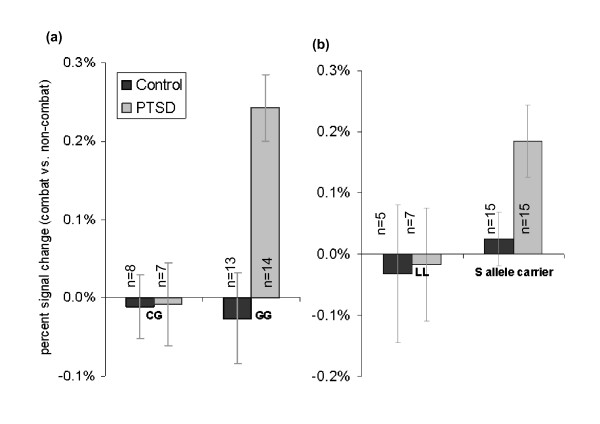
***SLC6A4 *(rs16965628) modulated the ventrolateral PFC in PTSD**. (a) mean activation level in the ventrolateral PFC ROI for combat vs. non-combat distractors presented during the working memory delay period was differentially modulated by rs16965628 in the PTSD group as compared to the trauma-exposed control group. (b) mean activation in the left amygdala ROI for combat vs. non-combat distractors presented during the working memory delay period was differentially modulated by 5-HTTLPR (S allele carrier, LL) in the PTSD group as compared to the trauma-exposed control group.

None of the genotypic variants showed evidence of Hardy-Weinberg disequilibrium in the present sample (see Table 2) or across race or diagnostic group in the parent sample of 387 subjects (data not shown). We found the serotonin transporter gene SNP rs16965628 significantly modulated activation of the ventrolateral PFC in the PTSD group, but not the non-PTSD group (see Figure 3). Specifically, ANCOVA modeling showed a significant effect of ventrolateral PFC activation during presentation of combat-distractors relative to non-combat distractor scenes in the working memory delay period [F(5,36) = 3.9, p_corr _< .05]. A significant diagnosis*genotype interaction was found in the ventrolateral PFC [F(1,36) = 7.8, p_corr _< .05] with planned comparisons revealing greater activation for GG genotypic variants with PTSD than trauma-exposed control participants [t(13) = 3.8, p = .0004] whereas no difference was observed between PTSD and trauma-exposed control participants with the CG genotype [t(25) = .05, p = .9]. The distribution of rs16965628 alleles were found to be non-independent with those of the 5-HTTLPR [χ^2^(2) = 8.2, p < .02]. This SNP was analyzed separately for each race. In the African American group, rs16965628 significantly modulated activation of the ventrolateral PFC in the PTSD group, but not the non-PTSD group [F(5,22) = 10.9, p < .0001]. Only a weak trend in this direction was observed in the European American group [F(5,22) = 1.7, p < .20].

Influence of 5-HTTLPR (biallelic) on the amygdala was examined in the context of reports showing effects in the right [30,31] and the left [28] amygdala while viewing threat-related cues. Our analysis of S allele carriers versus non-carriers (LL) showed a trend for left amygdala [F(6,35) = 3.4, p_corr _= .07] activation, but no association in the right amygdala [F(6,35) = 2.5, p_corr _> .2]. Planned comparisons showed greater left amygdala activation in the PTSD group than the trauma exposed control group for S allele carriers [t(28) = 2.2, p < .05] but no difference between diagnostic groups among participants with the LL genotype [t(28) = 0.1, p > .9] Activation in other ROIs, including ventrolateral PFC, fusiform gyrus, dorsolateral PFC, and lateral parietal cortex, did not attain the significance for biallelic 5-HTTLPR. The other hypothesized polymorphisms such as rs140701 and triallelic 5-HTTLPR did not attain significance for any of the ROIs (see Table 5).

With an insufficient sample of L_A_L_A _to perform between group analysis, the effects of L_A_L_A _on the overall group (PTSD + trauma-exposed) resulted in 9 subjects in the L_A_L_A _group and 33 subjects in the non-L_A_L_A _group (S and L_G _carriers). Significantly greater activation was present the L_A_L_A _group in the right amygdala [F(1, 37) = 5.86; p < .05], left amygdala [F(1, 37) = 5.98; p < .05], and the fusiform gyrus [F(1, 37) = 5.02; p < .05]. There were no between group differences in the ventrolateral PFC, the dorsolateral PFC, or the lateral parietal cortex.

## Discussion

The present study investigated the effects of serotonin transporter gene polymorphisms on neural activity associated with distraction from goal-directed cognitive processing by trauma-relevant cues in patients with PTSD. Brain activation was assessed during the delay interval between encoding and retrieval when active maintenance of information was disrupted by the presentation of trauma-related visual distractors that were irrelevant to the working memory task. We found rs16965628, the nearest tagging SNP downstream (3') of 5-HTTLPR (see Figure 1), significantly modulated task-related ventrolateral PFC activation in patients with PTSD while being distracted by combat compared to non-combat scenes (see Figure 3). In addition, the 5-HTTLPR showed trend level modulation of left amygdala activation during the working memory delay period in S allele carriers with PTSD (see Figure 3). We did not detect an association between triallelic 5-HTTLPR and task related neural activity in PTSD but confirmed greater bilateral amygdala activation associated with L_A_L_A _in the overall group.

We found the rs16965628 alleles were significantly associated with 5-HTTLPR alleles wherein the G allele of the SNP co-segregated with the S allele of 5-HTTLPR, which has been implicated in PTSD [11-15]. Interestingly, rs16965628 has been reported to exert the greatest relative effect amongst common variants on the level of serotonin transporter gene expression in human cell lines [56]. Its role was elaborated by assaying allelic imbalance in cell lines genotyped by the HapMap consortium. These investigators examined 55 SNPs in the 100 kb window around *SLC6A4 *to assess their influence on gene transcription. They found that aside from 5-HTTLPR, two SNPs rs16965828 and rs2020933 that are located in the first intron of the gene and highly correlated with each other, made the greatest contribution to the variation in serotonin transporter gene expression [56]. The present results suggest that rs16965628 accounts for a substantial difference in distractor-related activation of the ventrolateral PFC between PTSD and trauma-exposed control groups. The ventrolateral PFC is known to have an important role in the cognitive control and processing of emotionally salient information [42,43,45,46,68,69]. Previously, we reported greater activation in the PTSD group compared to the trauma-exposed control group in ventrolateral PFC during cognitive tasks such as working memory and executive processing [37,39,58]. Findings from our previous investigations [42,45] suggest an engagement of this region both in general emotion processing and in coping with emotional distraction. The observed intermediate phenotype of increased ventrolateral PFC activation during the distraction delay period appears to be related to the GG gentotpye of rs16965628 in patients with PTSD, which shows both increased emotional reactivity and a need for greater allocation of resources to maintain working memory performance in the face of emotional distraction. The observation of a significant association of a *SLC6A4 *SNP and PTSD (an association that has not previously been reported with a diagnostic phenotype) underscores that an intermediate phenotype approach may be more sensitive and powerful than behavioral measures given that neural circuitry is more proximal to gene effects than to behavior. The findings also highlight the potential value of intermediate phenotypes identified by imaging genetics for the discovery of associations between gene variants and disease.

Since rs16965628 has not been described in the imaging genetics literature, we consider our results in the context of closely associated 5HTTLPR [56]. A limited number of studies have examined the role of 5HTTLPR on the ventrolateral PFC, as most studies have focused on the amygdala. Surguladze and colleagues [34] reported that the S/S group showed greater functional connectivity between the right fusiform gyrus and the right ventrolateral PFC in response to fearful faces. Structural morphology of the ventrolateral PFC is associated with emotion-cognition interaction in carriers of the short allele of 5HTTLPR [70] who exhibit lower 5HT_1A _receptor density throughout the cortex [71]. In tasks of social cognition, 5HTTLPR modulates ventrolateral PFC [72]. This evidence considered together with increased ventrolateral activation in PTSD associated with emotion-cognition studies [37,57,58] and conventional symptom provocation studies [40,41,73] places the ventrolateral PFC at the nexus between 5HTTLPR and PTSD. Thus, increased vulnerability to PTSD and other disorders associated with 5HTTLPR genotype may be mediated through ventrolateral PFC engagement.

We find evidence at the trend level that 5-HTTLPR differentially modulates left amygdala activation in S allele carriers with PTSD. Specifically, S allele carriers with PTSD tended toward greater left amygdala activation in response to combat (relative to non-combat) distractors presented during the working memory delay period than trauma exposed controls. However this left amygdala activation difference was not observed between PTSD and trauma exposed control groups with the LL genotype. This finding is related to three lines of evidence showing that (i) 5-HTTLPR modulates threat-related amygdala activity in healthy normal subjects [28-35,74], (ii) heightened task-related amygdala activation in PTSD [37,38], and (iii) 5-HTTLPR may constitute a vulnerability for developing PTSD in the setting of trauma exposure [11]. Whereas initial reports in healthy subjects showed a 5-HTTLPR effect only in the right amygdala [30,31], subsequent reports extended this finding to the left amygdala [28,33]. The overall balance of neuroimaging data in PTSD from the past decade demonstrate greater amygdala activation in PTSD compared to controls [37,38,41,75-81]. These findings are consistent with the amygdala playing a central role in regulating responses to trauma reminders and cues [82]. Indeed, *SLC6A4 *has been implicated in PTSD, initially with data from biallelic 5-HTTLPR [11], and in more recent follow-up studies with triallelic 5-HTTLPR [11-15]. However, the present results concerning the role of 5-HTTLPR must be considered preliminary given the paucity of S as well as L homozygote's in our sample.

We did not observe the hypothesized association between 5-HTTLPR and right amygdala activation as previously reported in numerous imaging genetics investigations of healthy participants [28-34]. Several explanations may account for this difference. First, our sample contained the confounding effect of race. Second, the behavioral task in most of the prior studies consisted of viewing of fearful faces. This differs from the present working memory task with trauma related distractors that is designed to probe emotion-cognition interactions. Third, previous studies included healthy individuals that were not identified on the basis of trauma exposure and the present study does not compare trauma-exposed participants to healthy, non-exposed subjects. Finally, the threat-related nature of the previous task stimuli may elicit amygdala activation that has a unique association with 5-HTTLPR that is not specific to our working memory task where combat-related images are used as distractors.

We did not find any associations with triallelic 5-HTTLPR as might be suggested by several recent reports of association to PTSD diagnosis in the setting of high lifetime trauma exposure [11-15]. Our data was limited in assessing the effects of triallelic 5-HTTLPR on neural activity due to a lack of subjects possessing the L_A_L_A _gentoype. Reports of triallelic 5-HTTLPR generally show an interaction effect with the level of lifetime trauma exposure on diagnosis of PTSD whereas the present study was designed to match for level of trauma exposure between the PTSD and control group. Moreover, we are not aware of studies showing effect of triallelic 5-HTTLPR on brain function particularly as further modulated by exposure to childhood trauma. However in the overall group, we found increased left and right amygdala activity and fusiform gyrus activity associated with L_A_L_A_. These findings are consistent with results of emotion tasks eliciting greater amygdala activation that is differentially affected by the L_A_L_A _genotype in a normative sample [83] and in major depression [29].

While early imaging genetics studies of 5-HTTLPR assessed only amygdala activity, some recent studies in healthy subjects utilized cognitive attention and emotion processing tasks to show not just modulatation of amygdala, but also frontal cortical activation including the anterior cingulate, dorsolateral PFC, intraparietal sulcus, insula, and other regions [28,84]. We extend these findings by showing that rs16965628, the first tagging SNP downstream of 5-HTTLPR, modulates task ventrolateral PFC activation in PTSD associated with maintaining information in working memory while being distracted by combat pictures. Our findings support the supposition that fMRI data provides us with an intermediate phenotype that is closer to the function of proteins expressed by the candidate gene than a clinical entity. Thus, the definition of a precise intermediate phenotype that is closely linked to the biological function of gene expression is imperative. Core features of PTSD include hypervigilance and re-experiencing symptoms associated with the processing of emotional cues likely to be irrelevant to ongoing task demands, resulting in distractibility and compromised task performance. Emotional stimuli are known to influence behavioral performance on experimental tasks requiring cognitive processing [42,44-47], and therefore brain systems mediating cognitive control of emotion are relevant to PTSD [85]. While imaging phenotypes may be closer to the action of genes compared to behavioral or clinical phenotypes, it is certain that the imaging phenotypes employed in the present study are imprecise and are downstream manifestations of multiple gene systems working together to produce a complex ensemble of brain activity [27].

Based on preliminary nature of our results, the role of rs16965628 in PTSD deserves further investigation. While this SNP has not previously been described in association with PTSD, nor does data from our sample support an association of this SNP with PTSD as a diagnostic phenotype, there is currently insufficient information available to characterize the role of this SNP in PTSD or other anxiety disorders. Given the previous association of 5-HTTLPR with PTSD, a role for rs169656258 as a *modifier *is consistent with a number of other disorders, most notably cystic fibrosis, a monogenic disease determined by mutations of the cystic fibrosis transmembrane conductance regulator (*CFTR*) gene [86]. In cystic fibrosis, other genes are required to explain the clinical heterogeneity with the extent of liver [87] and lung [88] involvement not explained by *CFTR *alone. Conceived as a *modifier *SNP, the present results suggest that rs16965628 predicts brain activity related to the disruption of cognitive control by emotional or traumatic information in the ventrolateral prefrontal cortex. This type of model is certainly one that deserves to be investigated in PTSD where it is likely that multiple genes might predict onset of PTSD and other genes or SNPs within the causal genes might modify the variability of PTSD in concert with environmental exposures such as lifetime trauma.

It is important to consider the present findings in the broader context of neuroimaging and genetics findings observed in related neuropsychiatric disorders, particularly major depression and anxiety disorders. There is increasing evidence that a common set of underlying mechanisms are operating in depression and PTSD that may explain their shared diathesis [89]. Recent meta-analyses showed consistent patterns of amygdala hyperactivation in major depression [90], social phobia, specific phobia, and PTSD [91]. However, PTSD shows divergent findings when compared to the other anxiety disorders in the rostral anterior cingulate and ventral and dorsal medial prefrontal regions [91]. In these regions specific phobia and social phobia fail to show differences, while the PTSD literature contains evidence of both greater activation [[76], Lanius, 2002 #1016, Morey, 2008 #625, Pannu Hayes, 2009 #654] and lower activation [[92], Shin, 2005 #741, Bremner, 1999 #291, [93]] in ventromedial prefrontal cortex, which may be influenced by a variety of factors including illness chronicity, emotional versus trauma-specific stimuli, and others. Differences in ventrolateral PFC activity have been consistently demonstrated in both PTSD and depression. A meta-analysis of neuroimaging studies using emotional stimuli in depression found increased inferior frontal gyrus and left amygdala activation in response to negative emotional images [94].

Similarly, genetic evidence supports a shared diathesis for PTSD and depression. In 6,744 members of the Vietnam Era Twin Registry, major depression and PTSD showed a large genetic correlation (r = .77; 95% CI) and a modest individual-specific environmental correlation (r = .34; 95% CI) [95]. In addition, genetic influences common to depression explained 58% of the genetic variance in PTSD but only 15% of the total variance in risk for PTSD [95]. Individual-specific environmental influences common to depression explained only 11% of the variance in PTSD [95]. These data do not examine specific genetic loci nor the functional brain effects but are nevertheless suggestive of a shared pretrauma vulnerability.

## Limitations

Several limitations pose caveats to the interpretation of our results and warrant further investigation. Above all, despite the correction for multiple comparisons, the small sample size raises the possibility of Type I error. In general, our case-control design is susceptible to population stratification resulting from roughly equal samples of European American and African American ancestry with the latter having admixture from other races. We addressed this issue by verifying a lack of a race effect in the dependent variable and further by covarying for race in all statistical modeling. However, spurious associations can only be ruled out definitively by ascertaining a racially homogenous sample, increasing the sample size to permit separate analyses for both racial groups, or through the inclusion of a large number of ancestry informative markers in the analysis. Munafo and colleagues [35] have suggested minimum sample size on N = 70 for imaging genetics studies detecting 5HTTLPR effects. A much larger sample would also allow haplotype analyses of rs16965628 with 5-HTTLPR and other common polymorphisms, and consideration of epistatic effects. This would enable further analysis of polymorphisms in LD with rs16965628 including 5-HTTLPR and others that may be the major functional locus or loci. In spite of these limitations, we demonstrated reasonably robust effects perhaps because the imaging phenotype is closer to the effect of gene action than a behaviorally assessed clinical phenotype.

It is also possible that many of the effects that were significant at an uncorrected alpha level, but failed to reach the corrected significance level, might constitute Type II error resulting from the fairly small sample size. We attempted to match PTSD and control groups for level of trauma exposure, and a larger sample size and more sophisticated design would offer the ability to investigate whether gene-environment interactions (GxE) demonstrated on behavioral phenotypes may be detected on imaging phenotypes [51]. Gene effects may be better assessed by incorporating differences in environment and lifetime trauma exposure that interact to modulate gene expression as reflected by functional brain differences. Environmental and genetic modifiers have been studied in behavioral and psychiatric genetics studies of traumatic stress and PTSD [12,15,16,96], but GxE remains to be investigated in imaging genetics studies of PTSD where it could provide a window into functional brain differences and neuroplasticity that are modulated by the interaction of environmental and genetic factors.

## Conclusion

The *SLC6A4 *SNP rs16965628 and 5-HTTLPR are associated with a bias in neural circuit responses to traumatic reminders and cognitive control of emotions in patients with PTSD. Functional MRI may highlight dimensions of PTSD that are more closely related to susceptibility genes than current clinical categorizations, which are subjectively measured and rely on diagnostic criteria that are currently undergoing revision [97]. Neuroimaging may hold unique promise in highlighting specific functional brain differences as intermediate phenotypes to clarify links between genetic variation and disease phenotypes. Associations found with imaging genetics may guide further exploration and confirmation using conventional candidate gene associations with clinically defined phenotypes of PTSD.

## Conflicts of interests

The authors declare that they have no competing interests.

## Authors' contributions

RAM directed the project, contributed to the design, performed the data analysis and interpretation, wrote the manuscript, and obtained funding; ARH guided data analysis as well as synthesis and interpretation of genetic and neuroimaging data; ALG contributed to the analysis and interpretation of data; MAH directed the molecular genetics; HJM carried out the assays for genotyping; FD conceived and designed the study; GM conceived and designed the study and obtained funding. All authors contributed to writing and approval of the final manuscript.

## Pre-publication history

The pre-publication history for this paper can be accessed here:

http://www.biomedcentral.com/1471-244X/11/76/prepub
